# Slc39a2-Mediated Zinc Homeostasis Modulates Innate Immune Signaling in Phenylephrine-Induced Cardiomyocyte Hypertrophy

**DOI:** 10.3389/fcvm.2021.736911

**Published:** 2021-11-01

**Authors:** Yu Fang, Shun Wang, Jian Lv, Zhenyi Zhao, Ningning Guo, Gang Wu, Jingjing Tong, Zhihua Wang

**Affiliations:** ^1^Department of Cardiology, Renmin Hospital of Wuhan University, Wuhan, China; ^2^Shenzhen Key Laboratory of Cardiovascular Disease, Fuwai Hospital Chinese Academy of Medical Sciences, Shenzhen, China; ^3^State Key Laboratory of Cardiovascular Disease, Fuwai Hospital, National Center for Cardiovascular Diseases, Chinese Academy of Medical Sciences and Peking Union Medical College, Beijing, China; ^4^School of Pharmacy, Health Science Center, Shenzhen University, Shenzhen, China; ^5^School of Life Sciences, Central China Normal University, Wuhan, China

**Keywords:** SLC39A2, zinc homeostasis, cardiac hypertrophy, innate immune signaling, feedback circuit

## Abstract

Zinc dyshomeostasis has been involved in the pathogenesis of cardiac hypertrophy; however, the dynamic regulation of intracellular zinc and its downstream signaling in cardiac hypertrophy remain largely unknown. Using Zincpyr1 staining, we found a significant decrease of intracellular Zinc concentration in phenylephrine (PE)-induced hypertrophy of neonatal rat ventricular myocytes (NRVMs). We then screened SLC39 family members responsible for zinc uptake and identified Slc39a2 as the only one altered by PE treatment. Slc39a2 knockdown in NRVMs reduced the intracellular Zinc level, and exacerbated the hypertrophic responses to PE treatment. In contrast, adenovirus-mediated Slc39a2 overexpression enhanced zinc uptake and suppressed PE-induced Nppb expression. RNA sequencing analysis showed a pro-hypertrophic transcriptome reprogramming after Slc39a2 knockdown. Interestingly, the innate immune signaling pathways, including NOD signaling, TOLL-like receptor, NFκB, and IRFs, were remarkably enriched in the Slc39a2-regulated genes. Slc39a2 deficiency enhanced the phosphorylation of P65 NFκB and STAT3, and reduced the expression of IκBα. Finally, the expression of IRF7 was significantly increased by Slc39a2 knockdown, which was in turn suppressed by IRF7 knockdown. Our data demonstrate that zinc homeostasis mediated by a Slc39a2/IRF7 regulatory circuit contributes to the alteration of innate immune signaling in cardiomyocyte hypertrophy.

## Introduction

Cardiac hypertrophy is characterized by pathological growth of cardiomyocytes to compensate for the increased demand of contractile function under various cardiac stresses (1). Reprogramming of gene expression, energy metabolism and cell signaling has been involved in the pathogenesis of cardiac hypertrophy (1–4). As an essential element for fundamental biological functions, zinc plays vital roles in maintaining normal cell structure and function (5). Insufficient dietary zinc supplies or dysfunction of zinc handling proteins lead to intracellular zinc dyshomeostasis (6, 7), which has been associated with the pathophysiology of various cardiovascular diseases (8–11). In cardiac hypertrophy, the zinc level in serum is significantly reduced (8). Dietary Zinc depletion exacerbates the hypertrophic pathology in an obese mouse model (12). However, it remains elusive how intracellular zinc concentration is dynamically regulated during cardiac hypertrophy.

Intracellular zinc is tightly controlled by two families of zinc-specific transporters: the SLC30 (ZnT) family and the SLC39 (ZIP) family. The former functions to reduce the cytoplasmic zinc level, while the latter is responsible for zinc uptake (5, 13, 14). Whereas SLC30 proteins are more associated with metabolic disorders (14), SLC39 proteins are essential for maintaining normal cellular zinc concentration during development. Among all 14 SLC39 members identified in mouse, 7 genes show developmental abnormity after genetically inactivated (15). For instance, slc39a8-null mouse could not survive embryogenesis and exhibits left ventricular non-compaction as a result of excessive trabeculation and impaired myocardial compaction during heart development (16).

In cardiomyocytes, abnormal expressions of SLC39A7 and SLC30A7 coordinately mediate the mitochondria dysfunction and ER stress under hyperglycemia or doxorubicin insults (17). Du et al. (18) recently reported a crucial role of SLC39A2 in heart function recovery from ischemia/reperfusion injury. Its expression was induced by STAT3 signaling to compensate for the decrease of intracellular zinc concentration. However, whether and how SLC39A2 affects the development of cardiac hypertrophy remain unexplored.

Here we screened SLC39 family members in phenylephrine-induced cardiomyocyte hypertrophy, and identified SLC39A2 to be dynamically involved in the pathogenesis of hypertrophy. Transcriptome analysis unveiled a specific regulation of innate immune signaling. Our findings shed a light on the zinc-mediated signaling transduction in the development of cardiac hypertrophy and heart failure.

## Materials and Methods

### Animal Care

All experiments procedures were reviewed and approved by the Institutional Animal Care and Use Committee (IACUC) of Renmin Hospital of Wuhan University (No. 20180508) and performed in accordance with the guide for the care and use of laboratory animals published by National Institutes of Health, USA. All Sprague-Dawley rat were raised in a specific pathogen free environment (room temperature, 24 ± 3 °C; room humidity, 55 ± 5%) with a 12-h light/12-h dark cycle and fed normal chow. Male and female rats were bred in a ratio of 1:3, and the suckling rats were used for subsequent experiments.

### Cell Culture

Neonatal rat ventricular myocytes (NRVMs) were isolated and cultured as previously described (19). Briefly, the breast skin of Sprague-Dawley rat neonates (within 3 days) was disinfected with 75% ethanol. The chest skin was cut and the heart was extracted with bent tweezers and put in a culture dish (diameter 6mm) containing PBS. Cut off the large vessels attached to the surface of the heart, cut off the atrium, and fully cut into tissue debris. The fine-minced ventricular tissues were digested in an ADS buffer (NaCl 120 mM, HEPES (pH 7.4) 20 mM, NaH_2_PO_4_ 8 mM, glucose 6 mM, KCl 5 mM, MgSO_4_ 0.8 mM) containing 0.02% type II collagenase and 0.06% Pancreatin (Sigma, USA) at 37°C for 20 min/time for 7 times. Cardiomyocytes were separated from non-myocardial mesenchymal cells by density centrifugation using Percoll (GE Healthcare, USA). NRVMs were collected at the interface between 1.059 g/ml and 1.082 g/ml of Percoll solutions after centrifuge at 3000 rpm for 30 min at room temperature. NRVMs were seeded on 0.2% gelatin-coated culture dishes at 5 × 10^4^ cells/cm^2^ in Dulbecco's Modified Eagle Medium (DMEM) (Hyclone, Thermo Fisher Scientific, USA) containing 10% fetal bovine serum (FBS) (Gbico, Thermo Fisher Scientific, USA) for 24h. The medium was then replaced with DMEM containing 1% insulin-transferrin-selenium (ITS) (Cyagen Biosciences, USA) for another 24h before transfection or treatment. Phenylephrine (PE) (Abcam, UK) (50 μM) was administered for 48h to induce hypertrophy. ZnCl_2_ (10μM) and TPCA-1 (2μM), a dual inhibitor for STAT3 and NFκB (20), were administered to evaluate the impact of zinc supplement and NFκB pathway on cardiomyocyte hypertrophy. The siRNA sequence used for amplification was shown in Table 1.

**Table 1:** siRNAs designed for rat Slc39a2 and Irf7.

**Gene symbol**	**Forward primer**	**Reverse primer**
Slc39a2	UUAGAAAGAAUGUUUUGGUUACATG	CAUGUAACCAAAACAUUCUUUCUAAGU
Irf7	GCGCAACUGCCAUACUCCCAUCUTT	AGAUGGGAGUAUGGCAGUUGCGC

### WGA Staining

NRVMs used for WGA staining were cultured in a six-well plate. After cell culture and treatment, cells were washed with PBS and fixed with 4% paraformaldehyde for 15 min at room temperature. Then 0.2% Triton X-100 Cells were then permeabilized at room temperature for 10 min. 5.0 μg/mL WGA (Thermo Fisher Scientific, USA) was applied to incubate for 10 min at 37°C. For each step of treatment, the cells needed to be washed with PBS 3 times for 5 min each time. Then dye with DAPI for 10 min at room temperature. After dyeing with DAPI, washed 5–7 times with PBS for 5 min each time. Finally, the WGA staining pictures of cardiomyocytes were collected under a fluorescence microscope. Image Pro Plus 6.0 software was used to measure cardiomyocyte surface area (CSA). Each group measured more than 50 to calculate the surface area of cardiomyocytes.

### Zinc Measurement

When the cell culture was completed, Zn^2+^ measurements with fluorescent probes Free intracellular Zn^2+^ was monitored by loading the cells with Zinpyr-1 (5μM) in medium for 30 min at 37°C. Subsequently, discard the medium, wash 3–5 times with PBS, 5 min each time. Finally, an inverted microscope was used to observe and take pictures, and the results was statistically analyzed by Image J.

### Quantitative Real-Time PCR (qRT-PCR)

Fluorescence quantitative PCR was performed as previously described (11, 12). Total RNA was extracted from NRVM cells using Trizol reagent (TaKaRa, Shiga, Japan). RNA was quantified using NanoDrop (Thermo Fisher Scientific, USA). The first strand cDNA was synthesized using RevertAid First Strand cDNA Synthesis Kit (Thermo Fisher Scientific, USA), and SYBR Green PCR Master Mix (Monad, Suzhou, China) was used to perform fluorescent quantitative PCR. Finally, relative mRNA expression levels were analyzed by real-time qRT-PCR using LightCyclerR 480 (Roche, Switzerland). The primer sequence used for amplification was shown in Table 2. And the mRNA content of endogenous β-actin was set as a control to quantify the relative expression level of the target gene.

**Table 2:** Primers designed for qRT-PCR.

**Gene symbol**	**Forward primer**	**Reverse primer**
GAPDH	ACAGCAACAGGGTGGTGGAC	TTTGAGGGTGCAGCGAACTT
β-actin	CCCATCTATGAGGGTTACGC	TTTAATGTCACGCACGATTTC
Nppa	ATACAGTGCGGTGTCCAACA	AGCCCTCAGTTTGCTTTTCA
Nppb	CAGCTCTCAAAGGACCAAGG	GCAGCTTGAACTATGTGCCA
Slc39a1	GTCAGGCGCTAACCATGAAG	ACTCTTGCAAGGGGAACTGA
Slc39a2	GTTGCTGTGCTGGCTCATAA	AAAAACGTGCCAGCTGCTAT
Slc39a3	AGAAGGTCCTCTCCCTCTGT	AGAACCCCACCATCGTGAGG
Slc39a4	ATCCAAACAGCCCCATGAGA	CATGTGGATGAGAAGGCTGC
Slc39a5	CACACACAGGCTTCAGGAAC	GTCAGAGGAGCAACAGGACT
Slc39a6	CGTCCAGGCTCTGTTGAATG	TGAGTGGCACCAAGATGACT
Slc39a7	GTGCTCTTCCTCATCCCTGT	CACCACAAGAAAGGCGACAA
Slc39a8	GCTTGGATGATCACGCTCTG	CCCAACGTAGCAGGAACATG
Slc39a9	GGGATGTTACGTGGCTGGAA	TCACTGGCTTGGTGGTGTTT
Slc39a10	GGCAAACATAGGGGGCATCAG	GAGCATGCTGATGACTGTGG
Slc39a11	AGGGGTCATGTTAGCAGCTT	CCAAGCAGGGTCCAAGTTC
Slc39a12	GATCAGGCTTGCTTCTCTGC	CACTGCCACCGTGCTATAAC
Slc39a13	TTTTCTACAGCCCTGGGAGG	CTGTTGGGAGAGGGGGATTT
Slc39a14	AGGTCATCGTAGGCTCCATG	TCCCAGCTCGTGAGGAAATT
IRF7	CCTCTGCTTTCTGGTGATGC	GCGCTCAGTCATCAGAACTG

### Western Blot

Total protein was extracted from NRVM cells. NRVM cells lysed in Radio Immunoprecipitation Assay (RIPA) lysis buffers (Beyotime, Nanjing, China) for 30 min at 4°C. Protein concentration was determined using BCA assay followed by spectrophotometer readout at a wavelength of 562-nm. Equal quantities of each sample was fractionated on 12% SDS-PAGE gels. Resolved proteins were transferred to PVDF membrane (Millipore Corp, Bedford, MA, USA.) using a Trans-Blot apparatus (Bio-Rad, Hercules, CA, USA) at 200 mA for 100 min. Membranes were blocked with 5% skim milk for 1 h, washing 3 times with TBST, then incubated overnight at 4°C with primary antibodies. After incubation in primary antibody, the blots were probed with corresponding secondary antibody conjugated to horseradish peroxidase (Cell Signaling Technology, Inc). Detection of protein bands was developed using chemiluminescent Western detection system (Bio-Rad, Hercules, CA, USA), and analyzed using Quantity One software. The following Primary antibodies were used: SLC39A2, GAPDH were from Cell Signaling Technology, Inc.

### RNA Sequencing and Bioinformatics Analysis

Total RNA was extracted from NRVM cells transfected with siSlc39a2 or siNeg and in the absence or presence of PE. NRVM cells were used to extracted RNA according to the manufacturer's instructions. Transcriptome sequencing of RNA was completed by Beijing Genomics Institution (BGI). Three independent biological replicate samples were sequenced for each group. RNA-seq raw data and processed data have been uploaded to the GEO database. Accession code: GSE175884.

Normalization of expression matrix was accomplished by the normalize Between Arrays function in R. The different expression genes (DEGs) between siNeg, siNeg+PE and siSlc339a2+PE samples were screened using linear models for microarray data (limma) package. | log2 (fold change) | >1 and adjusted *P*-value < 0.05 were considered the threshold. Gene ontology (GO) and Kyoto Encyclopedia of Genes and Genomes (KEGG) pathway enrichment analysis of DEGs were carried out by using DAVID online tools. Gene Set Enrichment Analysis (GSEA) is used to screen significantly enriched signaling pathways and transcription factors with default parameters.

### Quantification and Statistical Analysis

Statistical analyses were performed by using GraphPad Prism 8 Software. All experimental data are presented as mean ± SEM of at least three independent replicates. Statistical significance for multiple comparisons was determined by one-way ANOVA or two-way ANOVA followed by Tukey's test. Bonferroni adjustment was used for *post hoc* analysis. Student's *t* test was used for comparisons between two groups. *P* < 0.05 was considered as statistically significant.

## Results

### Slc39a2 Is Associated With Zinc Depletion in Cardiomyocyte Hypertrophy

**Figure 1:**
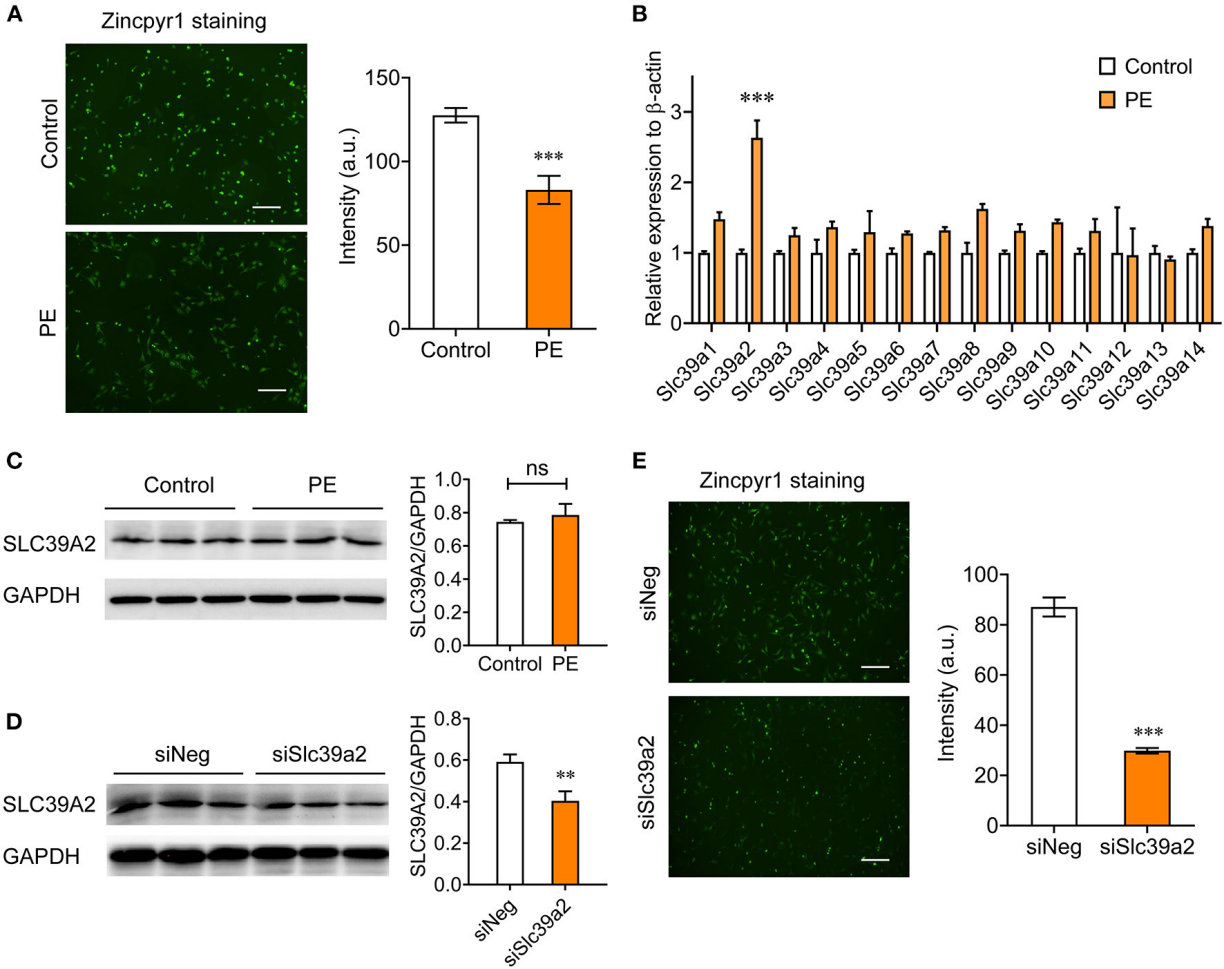
Abnormal expression of Slc39a2 is associated with zinc depletion in phenylephrine (PE)-treated cardiomyocytes. **(A)** Representative images (left) and quantification data (right) of Zincpyr1 staining in neonatal rat ventricular myocytes (NRVMs) with or without phenylephrine (PE; 50 μM) treatment. Scale bar, 200μm. ^***^P < 0.001 vs. Control. *n* = 3. **(B)** qRT-PCR analysis for Slc39a family members (1-14) in PE-induced cardiomyocyte hypertrophy. ^***^P < 0.001 vs. Control. *n* = 3. **(C)** Immunoblots (left) and quantification data (right) of SLC39A2 in NRVMs with or without PE treatment. *n* = 3. **(D)** Validation of knockdown efficacy of siSlc39a2. ^**^P < 0.01 vs. siNeg (negative control siRNA). *n* = 3. **(E)** Impact of Slc39a2 knockdown on Zinc contents in NRVMs. ^***^P < 0.001 vs. siNeg. *n* = 3.

We investigated the Zinc homeostasis in PE-induced cardiomyocyte hypertrophy using Zincpyr-1 staining, and found that PE treatment (50 μM) significantly reduced intracellular Zinc concentration in NRVMs (Figure 1A), suggesting a dysfunction of Zinc uptake. Thus, we screened the expression of all SLC39 family members by qRT-PCR. The results showed that Slc39a2 was the only member significantly induced by PE treatment (Figure 1B). However, the protein level of Slc39a2 was not affected by PE (Figure 1C), implicating a possible regulation of its transporter activity. Silencing Slc39a2 by siRNA in NRVMs significantly reduced intracellular Zinc concentration (Figures 1D,E). These data suggest that SLC39A2 might be involved in Zinc dyshomeostasis during cardiac hypertrophy.

### Slc39a2 Functions as an Anti-Hypertrophy Factor

**Figure 2:**
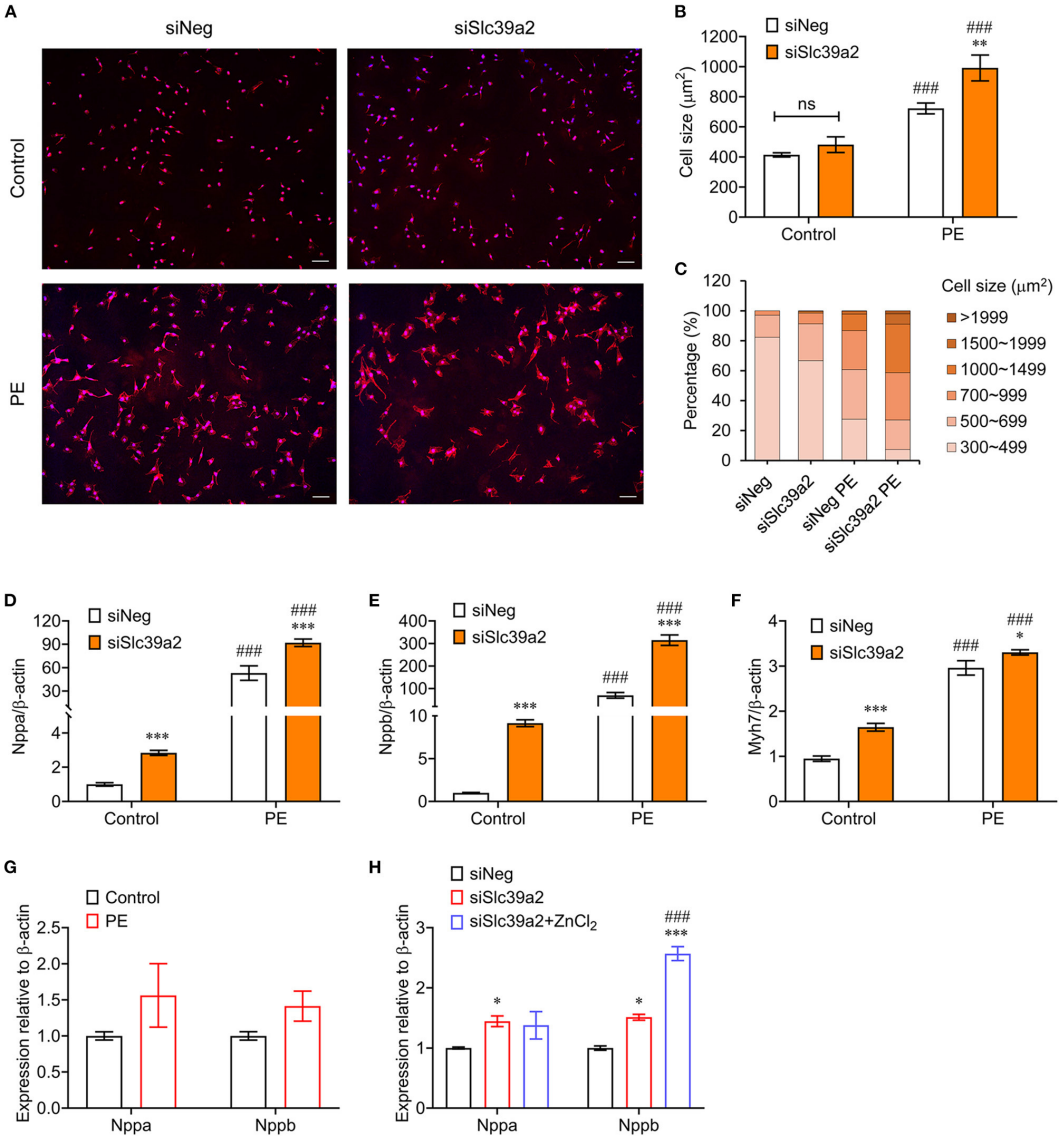
Slc39a2 deficiency exacerbates PE-induced cardiomyocyte hypertrophy. **(A)** Representative images of WGA staining showing the impact of Slc39a2 knockdown on PE-induced cell size enlargement. Scale bar, 100μm. **(B)** Averaged cell size measured by WGA staining. ^**^P < 0.01 vs. siNeg; ^*###*^*P* < 0.001 vs. Control. *n* = 3. **(C)** Counts of NRVMs with fragmented cell size range. **(D–F)** Impact of Slc39a2 on PE-induced hypertrophy markers, including Nppa **(D)**, Nppb **(E)**, and Myh7 **(F)**. ^*^P < 0.05, ^***^P < 0.001 vs. siNeg; ^*###*^*P* < 0.001 vs. Control. *n* = 3. **(G)** qRT-PCR analysis for Nppa and Nppb with and without ZnCl_2_ administration (10μM) in PE-treated NRVMs. *n* = 3. **(H)** Impact of zinc supplementation on NRVMs with or without Slc39a2 knockdown after PE treatment. ^*^P < 0.05, ^***^P < 0.001 vs. siNeg; ^*###*^*P* < 0.001 vs. siSlc39a2. *n* = 3.

We then examined the impact of SLC39A2 on cardiomyocyte hypertrophy. WGA staining showed that Slc39a2 knockdown did not change the cell size at basal level, but significantly potentiated the PE-induced cell size enlargement (Figures 2A–C). Moreover, Slc39a2 knockdown significantly increased the expression of hypertrophy-associated pathological genes, including natriuretic peptide A (Nppa; also known as Anf), natriuretic peptide B (Nppb; also known as Bnp), and myosin heavy chain 7 (Myh7; also known as β-MHC) at basal level, and further enhanced their induction by PE treatment (Figures 2D–F). These data suggest an anti-hypertrophic role of SLC39A2.

**Figure 3:**
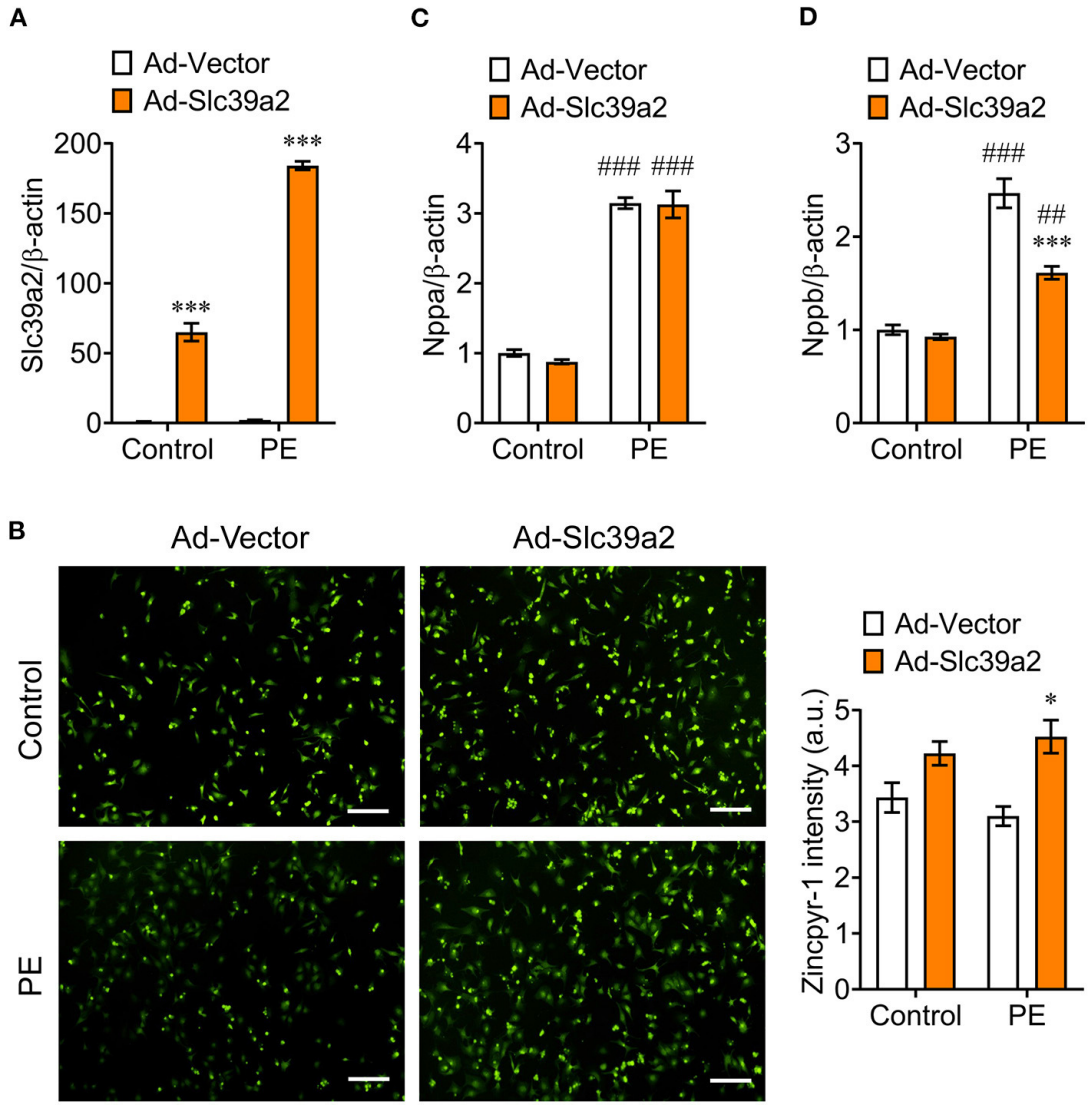
Slc39a2 overexpression increases zinc uptake and alleviates PE-induced cardiomyocyte hypertrophy. **(A)** Overexpression of Slc39a2 by adenovirus (Ad-Slc39a2). ^***^P < 0.001 vs. Ad-Vector. *n* = 3. **(B)** Representative images (left) and quantification data (right) showing the impact of Slc39a2 overexpression on Zinpry-1 staining in NRVMs with or without PE treatment. ^*^P < 0.05 vs. Ad-Vector. *n* = 3. **(C,D)** Impact of Slc39a2 overexpression on PE-induced expressions of Nppa **(C)** and Nppb **(D)**. ^***^P < 0.001 vs. Ad-Vector; ^*##*^*P* < 0.01, ^*###*^*P* < 0.001 vs. Control. *n* = 3.

We constructed an adenovirus expressing rat Slc39a2 to test its impact on cardiomyocyte hypertrophy (Figure 3A). Zincpyr-1 staining showed that Slc39a2 overexpression significantly increased the intracellular zinc concentration (Figure 3B), and suppressed the PE-induced expression of Nppb, but not Nppa (Figures 3C,D). These data suggest that SLC39A2-mediated zinc uptake plays a protective role in cardiomyocyte hypertrophy.

### Slc39a2 Deficiency Potentiates the Transcriptome Reprogramming Induced by PE

**Figure 4:**
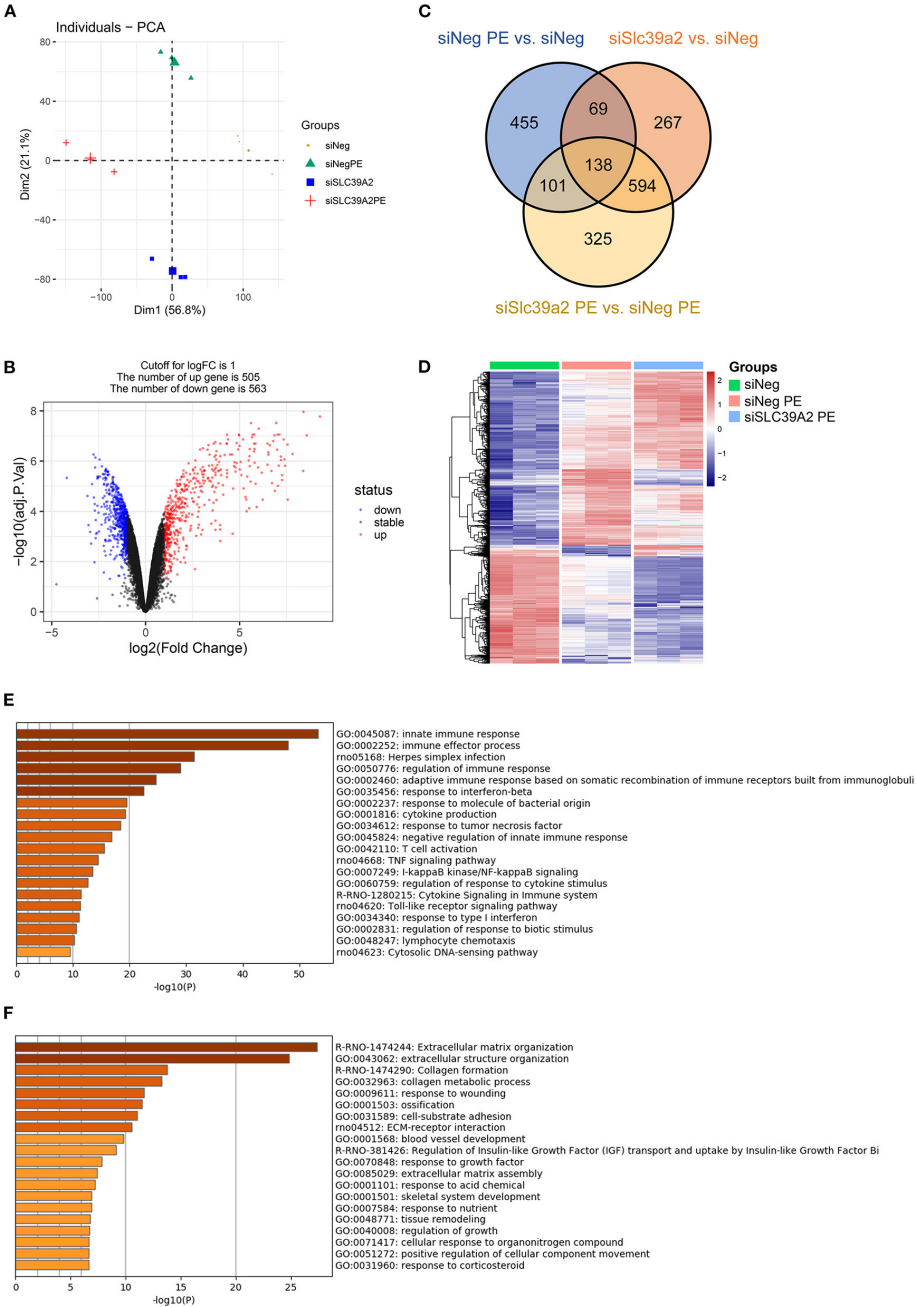
Slc39a2 deficiency potentiates the transcriptome reprogramming induced by PE. **(A)** Principal component analysis (PCA) from the RNA-seq data showing the overall impact of Slc39a2 knockdown on the transriptome of NRVMs with or without PE treatment. **(B)** Volcano mapping showing the number of up- and down-regulated genes (≥ 2 folds) by Slc39a2 knockdown. **(C)** Venn map showing the relationship of differentially expressed genes among 3 comparisons (siNeg PE vs. siNeg; siSlc39a2 vs. siNeg; siSlc39a2 PE vs. siNeg PE). **(D)** Heatmap showing the impact of siSlc39a2 on PE-induced transcriptome reprogramming. **(E,F)** Gene Ontology (GO) analyses of up-regulated **(E)** and down-regulated **(F)** genes by siSlc39a2 measured by Metascape.

To explore the underlying mechanism, we performed RNA-seq using siNeg- or siSlc39a2-transfected NRVMs with and without PE treatment. Principal component analysis (PCA) showed that Slc39a2 deficiency established a unique gene expression pattern different from that observed in PE-induced hypertrophy (Figure 4A). After Slc39a2 knockdown, 505 genes were up-regulated while 563 genes were down-regulated (|log2(fold change)| ≥ 1; Figure 4B). A subset of these genes (207 genes) was found to be differentially regulated by PE treatment (Figure 4C). Heatmap analysis showed that genes related to PE treatment were largely potentiated after Slc39a2 knockdown (Figure 4D), suggesting a suppressive role of Slc39a2 in hypertrophic transcriptome reprogramming. Gene Ontology (GO) analysis showed that the up-regulated genes after Slc39a2 knockdown were mostly related to innate immune response (Figure 4E), whereas the down-regulated were functionally associated with extracellular matrix organization (Figure 4F).

### Slc39a2 Deficiency Activates Innate Immune Signaling During Cardiomyocyte Hypertrophy

**Figure 5:**
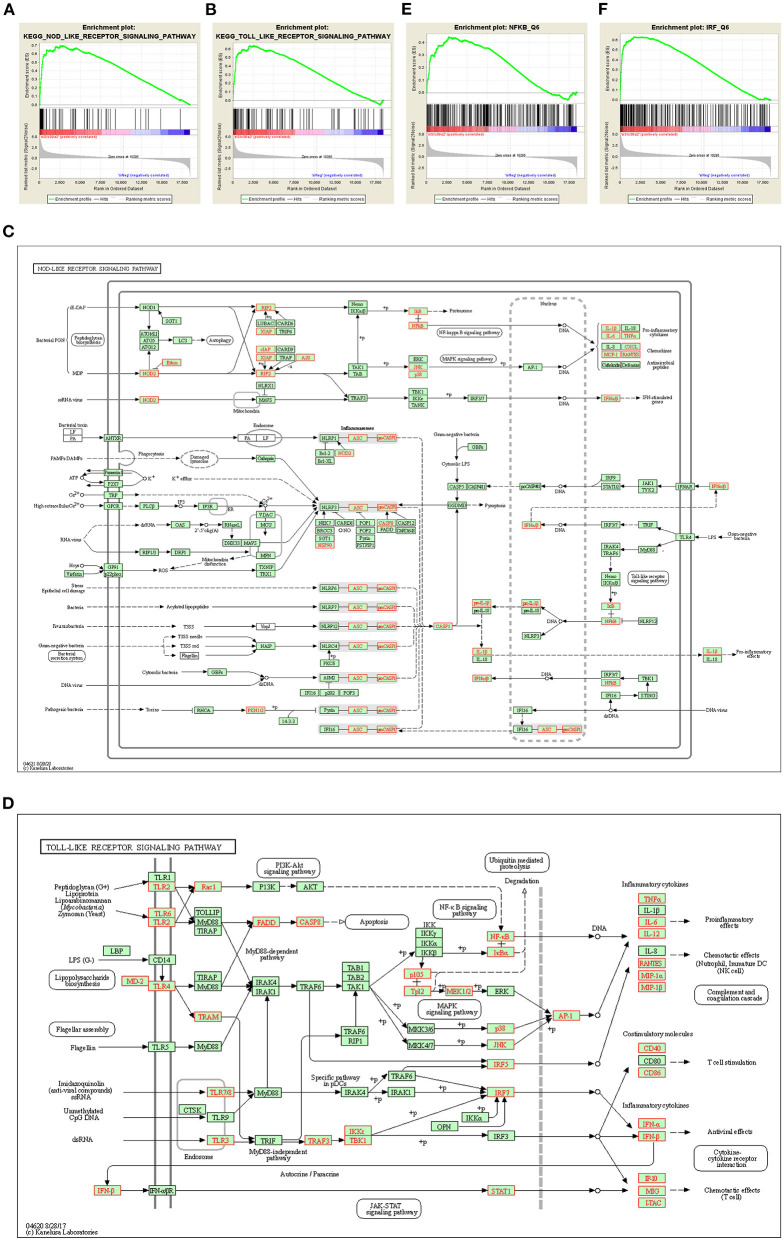
Slc39a2 deficiency activates innate immune signaling during cardiomyocyte hypertrophy. **(A,B)** Gene set enrichment analysis (GSEA) identified NOD-like receptor **(A)** and TOLL-like receptor **(B)** pathways to be significantly enriched by Slc39a2 knockdown. **(C,D)** KEGG analysis showing the siSlc39a2-affected genes involved in NOD-like receptor **(C)** and TOLL-like receptor **(D)** pathways. **(E,F)** GSEA identified NFκB and IRF as key factors involved in siSlc39a2-mediated transcriptome reprogramming.

Gene set enrichment analysis (GSEA) identified NOD-like receptor (Figure 5A) and TOLL-like receptor (Figure 5B) pathways to be remarkably enriched among Slc39a2-regulated genes. These genes covered most of the steps in both of the two signaling pathways, eventually leading to the activation of pro-inflammatory factors, including nuclear factor kappa B (NFκB) and interleukin 6 (IL-6) (Figures 5C,D). This was further confirmed by the observation that NFκB was significantly enriched from the regulator analysis of GSEA (Figure 5E). Interestingly, interferon regulatory factors (IRFs) were also profoundly enriched (Figure 5F).

**Figure 6:**
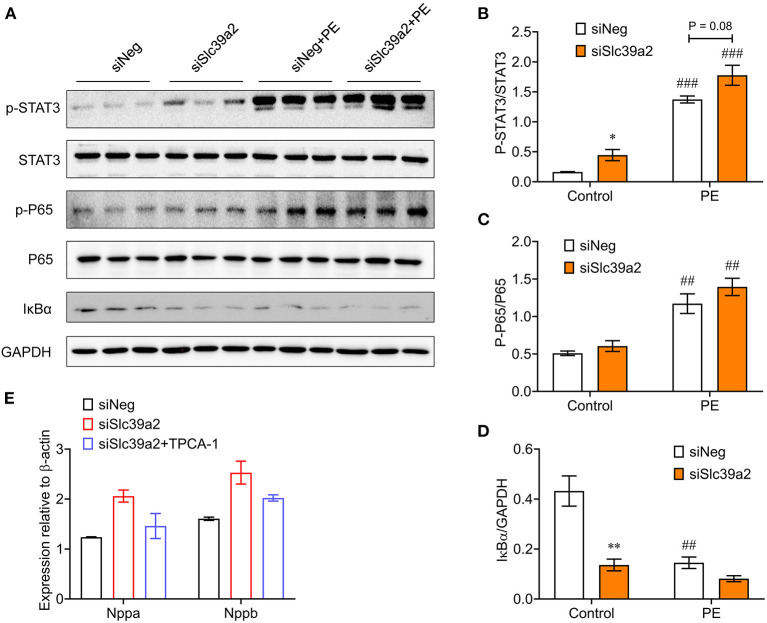
Slc39a2 deficiency enhances IκBα degradation and NFκB/STAT3 phosphorylation. **(A)** Immunoblots of phosphorylated STAT3, total STAT3, phosphorylated P65, total P65, IκBα, and GAPDH in siNeg- or siSlc39a2-transfected NRVMs with and without PE treatment. **(B-D)** Quantification data of p-STAT3/STAT3 ratio **(B)**, p-P65/P65 ratio **(C)**, and IκBα/GAPDH ratio **(D)**. ^*^P < 0.05, **P < 0.01 vs. siNeg; ^*##*^*P* < 0.01, ^*###*^*P* < 0.001 vs. Control. *n* = 3. **(E)** Impact of TPCA-1, an inhibitor of NFκB (2μM), on Nppa and Nppb expressions in NRVMs with or without Slc39a2 knockdown after PE treatment. *n* = 3.

To validate the activation of innate immune signaling pathways, we performed Western blot analysis for the phosphorylations of P65 NFκB and signal transducer and activator of transcription 3 (STAT3), a downstream effector of IL-6. Both of them were significantly enhanced in PE-treated NRVMs (Figures 6A–C). Consistently, the expression of inhibitor of NF-kappa B alpha (IκBα) was significantly reduced (Figures 6A,D). At basal level, Slc39a2 knockdown significantly increased STAT3 phosphorylation and IκBα expression (Figures 6A–D). Though not approaching statistical significance, Slc39a2 knockdown showed a trend to enhance the PE-induced STAT3 phosphorylation, P65 phosphorylation, and IκBα expression (Figures 6A–D). Administration of TPCA-1 (2μM), a dual inhibitor for STAT3 and NFκB (20), showed a trend to reverse the effect of Slc39a2 knockdown on Nppa and Nppb expression after PE treatment, but did not approach statistical significance (Figure 6E), suggesting that NFκB pathway partially mediates the effects of Slc39a2 on hypertrophy.

### Interplay Between Slc39a2 and Irf7 During Cardiomyocyte Hypertrophy

**Figure 7:**
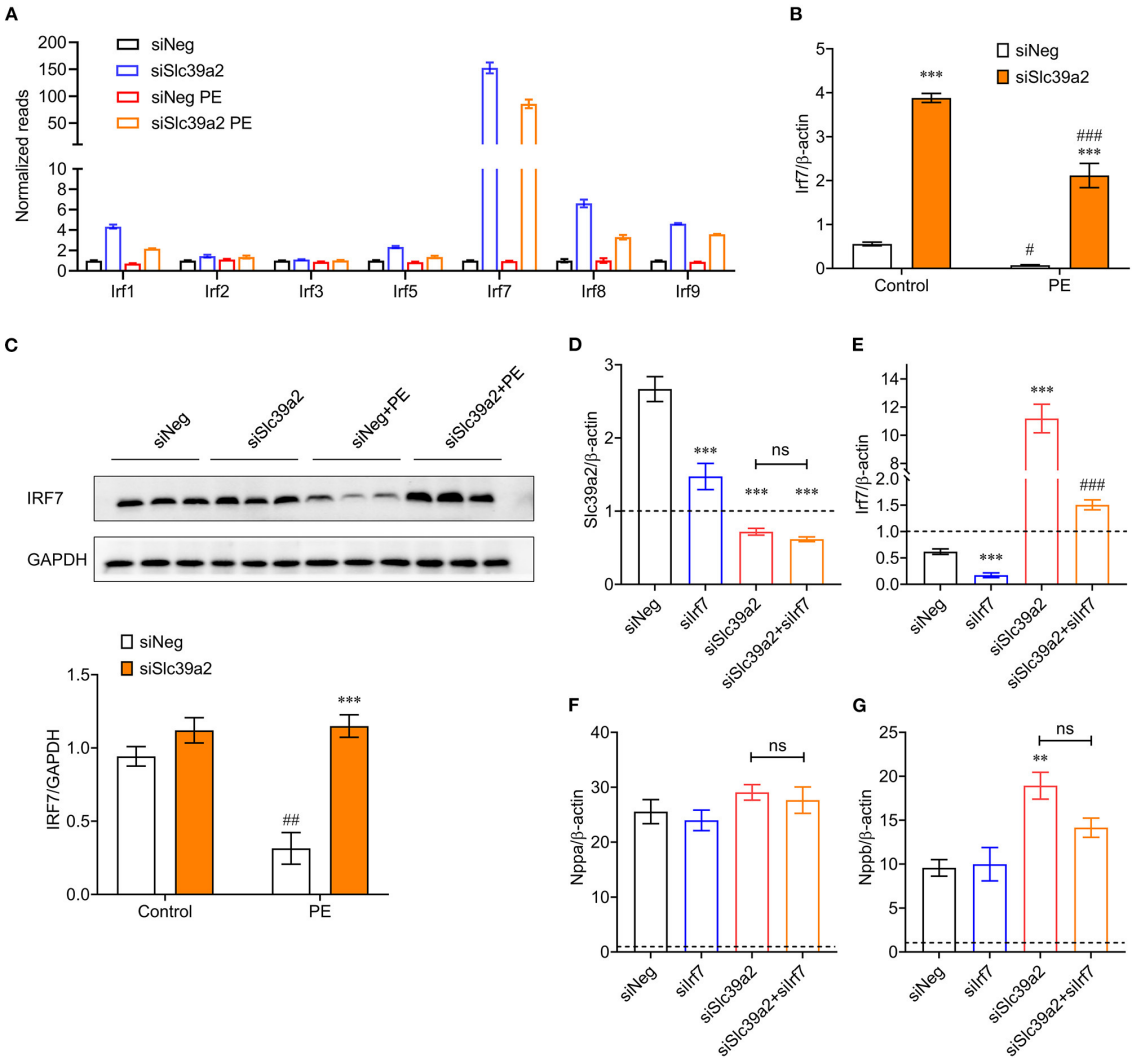
Interplay between SLC39A2 and IRF7 during cardiomyocyte hypertrophy. **(A)** Reads of IRF family genes (Irf1-9) from RNA-seq data. *n* = 3. **(B)** Validation of Irf7 expression by qRT-PCR. ^***^P < 0.001 vs. siNeg; ^#^*P* < 0.05, ^*###*^*P* < 0.001 vs. Control. *n* = 3. **(C)** Immunoblots (upper) and quantification data (lower) for IRF7 expression at protein level in NRVMs with or without Slc39a2 knockdown and PE treatment. ^***^P < 0.001 vs. siNeg; ^*##*^*P* < 0.01 vs. Control. *n* = 3. **(D,E)** Validation of knockdown efficacy siSlc39a2 **(D)** and siIrf7 **(E)** in PE-treated NRVMs. Data were normalized to control group (dotted line). ^***^P < 0.001 vs. siNeg; ^*###*^*P* < 0.001 vs. Control. *n* = 3. **(F,G)** Impact of combined knockdown of Slc39a2 and Irf7 on hypertrophy markers, Nppa **(F)** and Nppb **(G)**. ^**^P < 0.01 vs. siNeg. *n* = 3.

Since IRFs were dramatically enriched in Slc39a2-associated transcriptome reprogramming, we screened the expression of all IRF members (1–9), among which Irf7 stood out in response to Slc39a2 deficiency (Figure 7A). qRT-PCR analysis showed that the expression of Irf7 was significantly suppressed by PE treatment, but largely reversed after Slc39a2 knockdown (Figure 7B). Consistently, Western blot analysis also detected an upregulation of IRF7 expression at protein level after Slc39a2 knockdown (Figure 7C), suggesting an inhibitive effect of Slc39a2 on IRF7 expression. Interestingly, knockdown of Irf7 in NRVMs significantly suppressed the expression of Slc39a2 (Figure 7D), implicating a positive regulation of Slc39a2 by Irf7. Thus, when the function of SLC39A2 is suppressed in cardiomyocyte hypertrophy, the induction of Irf7 might contribute to the increased transcription of Irf7. However, Irf7 knockdown did not alter the expression of pathological genes, nor did it block the pro-hypertrophic effect of siSlc39a2 (Figures 7E–G). These data suggest a regulatory circuit between Slc39a2 and Irf7.

## Discussions

The pathogenesis of pathological cardiac hypertrophy is a programmed process coordinated by regulations at multiple layers, such as reprogramming of gene expression, energy metabolism and signal transduction (2–4, 21, 22). Our data demonstrate that Slc39a2-mediated Zinc homeostasis contributes to the remodeling of innate immune signaling in cardiomyocyte hypertrophy.

Zinc plays a pivotal role in signal transduction; however, the detailed mechanism remains largely unexplored (10). Its dyshomeostasis has been associated with the pathophysiology of various cardiovascular diseases (23, 24). In cardiac hypertrophy, the zinc level in serum is significantly reduced (8). In line with this report, we find that PE treatment significantly lowers intracellular zinc concentration in NRVMs (Figure 1A). Lack of intracellular zinc mediated by Slc39a2 knockdown aggravates cardiomyocyte hypertrophy (Figure 1E and Figure 2). These data suggest a crucial role of Zinc in maintenance of cell physiological function.

SLC39 family transporters are responsible for the uptake of zinc from the extracellular environment (18). Zinc concentration is remarkably lowered by Slc39a2 knockdown in NRVMs (Figure 1E), indicating that SLC39A2 contributes to zinc uptake in cardiomyocytes. Hypertrophic stimulation increases Slc39a2 at the mRNA level, but not at the protein level (Figures 1B,C). Considering the fact that zinc concentration is reduced in PE-treated NRVMs, our findings suggest a possible inhibition of SLC39A2 transporter activity during hypertrophy that subsequently leads to a compensatory activation of its transcription. Importantly, inhibition of Slc39a2 induces Irf7 expression, which in turn activates Slc39a2 expression (Figure 7). Thus, IRF7 function as the feedback regulatory factor to govern Slc39a2 expression in response to its activity. Negative feedback regulation has been referred as a fundamental mechanism to maintain the cell homeostasis, while the detailed biological process remains unclear. Our study provides a vivid example showing how this regulatory circuit can be realized. Since a number of transcription factors are zinc finger proteins, the transcriptional suppression of IRF7 by Slc39a2 might be attributed to a zinc finger transcription suppressor, which requires further screening studies to determine its identity.

Emerging evidence reveals an important role of innate immune and inflammatory signaling processes in cardiovascular systems (25). Toll-Like receptor and NOD-Like receptor signaling pathways are both classical innate immune signaling pathways. As the central transcription effector, IRFs mediates the pro-inflammatory gene transcription by activated innate immune signaling (26). Through a series of genetic modified mouse models, Jiang et al. (27–29) demonstrated that IRF7, IRF8, and IRF9 were anti-hypertrophy factors consistently down-regulated in cardiac hypertrophy and heart failure. Although these factors play exhibit similar regulation and function in cardiac hypertrophy, they mechanistically carry out their function via different mechansims. IRF7 inactivates NFκB through directly interacting with inhibitor of kappaB kinase-beta (IκBβ) (29). IRF8 directly interacts with nuclear factor of activated t cells 1 (NFATc1) to prevent its translocation intro nucleus and thus inhibits the hypertrophic response (27). Whereas IRF9 competes with p300 for binding to the transcription activation domain of myocardin, a coactivator of serum response factor (SRF), and thus suppresses its transcriptional activity at CArG box-dependent reporters (28). In the present study, we find that the expression of IRF7 is significantly suppressed after PE treatment; however, silencing IRF7 has no influence on PE-induced hypertrophy markers (Figures 7F,G). A possible reason is that the role of IRF7 in cardiac hypertrophy is a combination of its function in both cardiomyocytes and non-cardiomyocytes in the heart.

The nuclear factor NFκB has coevolved with the IRFs family (30). It is activated by a kinase family IκB kinases (IKKs), which phosphorylates IκBα followed by degradation and releases its inactivation effect on NFκB (30–33). Activation of NFκB contributes to the pathological remodeling of the heart during hypertrophy (34–38). Consistent with our observation, Ho et al. (39) found that supplementation of dietary zinc inhibits NFκB signaling pathway and the downstream inflammation in diabetic cardiomyopathy. In response to numerous cytokines and chemokines, STAT3 converges multiple signal transduction pathways and contributes to myocardial remodeling and fibrosis in cardiac hypertrophy (40, 41). Du et al. (18) reported that Slc39a2 is up-regulated via STAT3 upon reperfusion from myocardial infarction. This is consistent with a previous study that Slc39a6 (also known as LIV1) is a downstream target of STAT3 functioning in epithelial-mesenchymal transition (42). Here we find that STAT3 might also be a downstream target of SLC39A2-mediated zinc signaling (Figure 6), suggesting an interplay between zinc and STAT3 signaling. Nonetheless, how zinc directly modify NFκB and STAT3 pathways need further investigation.

It is surprising that the protein level of Slc39a2 does conform to its mRNA changes. One possible reason is the ribosome selectivity that may require more mRNA content of Slc39a2 to maintain a constant translation rate. The other reason is a potential accelerated protein degradation process during cardiac hypertrophy. Further experiments are needed to clarify the detailed mechanisms. Although we did not observe expression changes in other SLC39 members except Slc39a2, slc39a8, slc39a12, and slc39a14 have been associated with cardiovascular dysfunctions (16, 43, 44). It needs further exploration how different SLC39 members coordinate to maintain normal zinc homeostasis under pathophysiological conditions.

Taken together, our study unveils the molecular mechanism for the dynamic regulation of Slc39a2 during cardiac hypertrophy, and demonstrates that SLC39A2-mediated zinc homeostasis contributes to the remodeling of innate immune signaling in cardiomyocyte hypertrophy. Leveraging Slc39a2 to target intracellular zinc metabolism might be a novel strategy to treat cardiac hypertrophy and heart failure.

## Data Availability Statement

The datasets presented in this study can be found in online repositories. The names of the repository/repositories and accession number(s) can be found in the article/supplementary material.

## Ethics Statement

The animal study was reviewed and approved by Ethical Committee of Renmin Hospital of Wuhan University.

## Author Contributions

ZW, JT, and GW conceived and supervised the project. YF and SW performed most of the cell and molecular experiments with inputs from NG and ZZ. JL performed the bioinformatic analyses. YF, SW, and ZW analyzed the data and drafted the manuscript. All authors have access to the original data and approved the publication of the manuscript.

## Funding

This study was supported by funds from National Natural Science Foundation of China (Nos. 81722007, 82070231, 81800085, 81870301, and 32170763), National Health Commission of China (No. 2017ZX10304402001-008), and start-up funds from State Key Laboratory of Cardiovascular Disease Fuwai Hospital Chinese Academy of Medical Sciences, Shenzhen.

## Conflict of Interest

The authors declare that the research was conducted in the absence of any commercial or financial relationships that could be construed as a potential conflict of interest.

## Publisher's Note

All claims expressed in this article are solely those of the authors and do not necessarily represent those of their affiliated organizations, or those of the publisher, the editors and the reviewers. Any product that may be evaluated in this article, or claim that may be made by its manufacturer, is not guaranteed or endorsed by the publisher.

## Abbreviations

GO: Gene ontology
GSEA: Gene Set Enrichment Analysis
IL-6: interleukin 6
IRF: interferon regulatory factor
IκBα: inhibitor of NF-kappa B alpha
IκBβ: inhibitor of kappaB kinase-beta
IKK: IκB kinases
KEGG: Kyoto Encyclopedia of Genes and Genomes
MEF: mouse embryonic fibroblast
Myh7: myosin heavy chain 7
NFATc1: nuclear factor of activated t cells 1
NRVM: neonatal rat ventricular myocyte
Nppa: natriuretic peptide A
Nppb: natriuretic peptide B
NFκB: nuclear factor kappa B
PE: Phenylephrine
PCA: Principal component analysis
STAT3: Signal transducer and activator of transcription 3
WGA: Wheat Germ Agglutinin.
